# Limited awareness and use of HIV post‐exposure prophylaxis among people vulnerable to HIV acquisition in Western Kenya: a cross‐sectional analysis

**DOI:** 10.1002/jia2.26472

**Published:** 2025-06-26

**Authors:** Glenna Schluck, Matthew L. Romo, Josphat Kosgei, Michael C. Thigpen, Natalie Burns, Rael Bor, Deborah Langat, Christine Akoth, Adam Yates, Curtisha Charles, Haoyu Qian, Britt Gayle, Margaret Yacovone, Fredrick Sawe, Trevor A. Crowell, for the Multinational Observational Cohort of HIV and other Infections (MOCHI) Study Group

**Affiliations:** ^1^ Henry M. Jackson Foundation for the Advancement of Military Medicine Bethesda Maryland USA; ^2^ U.S. Military HIV Research Program, CIDR Walter Reed Army Institute of Research Silver Spring Maryland USA; ^3^ U.S. Military HIV Research Program Walter Reed Army Institute of Research ‐ Africa Kericho Kenya; ^4^ HJF Medical Research International Kericho Kenya; ^5^ National Institute of Allergy and Infectious Diseases National Institutes of Health Rockville Maryland USA

**Keywords:** post‐exposure prophylaxis, pre‐exposure prophylaxis, HIV, sexually transmitted infection, health risk behaviours, implementation science

## Abstract

**Introduction:**

HIV post‐exposure prophylaxis (PEP) can prevent HIV acquisition and facilitates linkage to pre‐exposure prophylaxis (PrEP) for people with ongoing vulnerability. We assessed PEP awareness and use in Western Kenya.

**Methods:**

We used cross‐sectional screening/enrolment data from the Multinational Observational Cohort of HIV and other Infections (MOCHI) study. Eligible participants had behavioural vulnerability to HIV and were ages 14–55 years. Participants completed questionnaires on demographics, sexual/behavioural history, and PEP/PrEP awareness and use. Depression was assessed using the Patient Health Questionnaire (PHQ‐9) with none/minimal, mild and moderate/severe depression defined as PHQ‐9 scores of 0–4, 5–9 and ≥10, respectively. We used multivariable robust Poisson regression with purposeful variable selection to estimate adjusted prevalence ratios (aPRs) and 95% confidence intervals (CIs) for factors associated with PEP awareness.

**Results:**

From December 2021 to May 2023, 398 participants indicated whether they heard of PEP. The median age was 22 years (IQR 19–24), 316/399 (79.2%) were female and 315/389 (81.0%) reported sex work or transactional sex. One hundred fourteen (28.6%) participants had never heard of PEP, of whom 79 (69.3%) had also not heard of PrEP. Among 284 participants who had heard of PEP, 74 (26.1%) did not know where to access it. Seventy‐one participants (17.8%) had taken PEP, of whom 17 (23.9%) encountered problems accessing PEP such as unavailability (*n* = 5) or prohibitive expense (*n* = 4). In the final model, only <12 years of education (aPR 1.65 [95% CI 1.16–2.34]) and not cohabitating (aPR 2.81 [95% CI = 1.11–7.08]) were associated with never having heard of PEP. Among participants who had heard of PEP, factors associated with not knowing where to access PEP were <12 years of education (aPR 2.20 [95% CI 1.37–3.54]) and depression (mild aPR 1.86 [95% CI 1.17–2.96]; moderate/severe aPR 1.84 [95% CI 1.09–3.09], compared to none/minimal).

**Conclusions:**

Despite enrolling a behaviourally vulnerable group potentially eligible for PEP, we identified substantial gaps in PEP awareness, access and use. Demand generation and improved access to PEP are needed to maximize the impact on reducing HIV incidence. Interventions to improve PEP awareness and access may be most impactful for people with lower education or when coupled with mental health services.

## 1 INTRODUCTION

HIV post‐exposure prophylaxis (PEP) is broadly recommended to prevent HIV acquisition after occupational or non‐occupational exposures [1, 2, 3, 4, 5, 6]. Prompt administration after exposure and completion of an adequate course are crucial to prevent the establishment of persistent infection. Guidelines recommend initiation of a 28‐day course of PEP ideally within 24 hours but no later than 72 hours after an HIV exposure [7, 8, 9, 10].

PEP provides a prevention option for people who experience unanticipated intermittent HIV exposures, which includes when other methods fail, such as condom breakage or pre‐exposure prophylaxis (PrEP) interruptions due to pill fatigue or stockouts. Completing a PEP course is a potential entry point for PrEP (and/or the use of other prevention tools), particularly for individuals anticipating ongoing exposures that have the potential for HIV transmission [11, 12].

PEP has been largely underutilized in the global HIV response [13]. In Kenya, PEP is primarily available in hospitals and HIV clinics and is recommended in local guidelines for individuals without HIV within 72 hours of an exposure that poses a significant risk of HIV transmission [14]. The introduction and increasing use of oral tenofovir‐based PrEP in African countries [15] is changing the landscape of HIV prevention, with additional PrEP options on the horizon. Nevertheless, PEP maintains an important and complementary role to PrEP as the only biomedical prevention tool that can be used *after* a potential HIV exposure. Therefore, understanding gaps in PEP awareness and access may inform strategies to maximize impact on reducing HIV incidence.

In this paper, we assessed and characterized PEP awareness and use in people with behavioural vulnerability to HIV in Western Kenya and determined socio‐demographic and behavioural factors associated with lack of PEP awareness, access and use.

## 2 METHODS

### 2.1 Participant recruitment and eligibility

For these analyses, we used cross‐sectional data from the screening and enrolment visits at the Kericho and Homa Bay, Kenya sites of the Multinational Observational Cohort of HIV and other Infections (MOCHI; Clinicaltrials.gov NCT05147519). Kericho is located alongside trucking routes with bars and other venues known to be associated with commercial sex work. Homa Bay is a fishing community with bars known to be associated with commercial sex work, a “fish for sex” trade between fishermen and fishmongers, and an established community of men who have sex with men (MSM) [16]. Recruitment occurred in bars, clubs and other venues frequented by populations with high HIV incidence. A community engagement team worked with venue owners to facilitate approaching potential participants and secure on‐site space for private briefings. In Homa Bay, the community engagement team also recruited at fish markets. Potential participants were referred to a study site for screening and referrals within social networks were encouraged. Eligible participants were 14–55 years old, not living with HIV, and considered vulnerable to HIV and other sexually transmitted infections (STIs) based on one or more of the following criteria in the previous 24 weeks: (1) documented history of a newly diagnosed STI (confirmed through participant‐provided medical record review); (2) self‐reported intercourse in exchange for money as a regular source of income; (3) self‐reported condomless vaginal or anal intercourse with at least three partners living with HIV or of unknown status; (4) self‐reported injection drug use; and (5) self‐reported MSM status. Participants were excluded if they had a positive urine pregnancy test, reported participation in an HIV vaccine study with receipt of an active product, or had any condition or substance use that could interfere with their safe participation in the study.

### 2.2 Ethical considerations

All participants provided written informed consent in English or Kiswahili prior to any study procedures. Assent and parental consent were required for 14‐year‐old participants [17]. Illiterate participants were consented with an adult impartial witness present. The study was approved by institutional review boards at the Kenya Medical Research Institute, the Walter Reed Army Institute of Research and all collaborating institutions.

### 2.3 Socio‐demographic and behaviour data collection and categorization

Participants completed questionnaires related to demographic characteristics and recent behaviours at screening for study eligibility within 7 days of enrolment. Questionnaires were administered primarily via computer‐assisted self‐interview (CASI).

Demographic characteristics included enrolment site, sex, age, marital status, education level, occupation, weekly household income and the distance from the participant's home to the clinic. Age was dichotomized with a cut‐off of 24 years as people under 24 are at an increased risk for HIV acquisition [18]. To maintain consistency with other work from our group [19], weekly household income and distance from the clinic were dichotomized at the 20th percentile. For weekly household income, this allows for comparisons between participants from the poorest households with others. For weekly household income, this allows for comparisons between participants who lived closest to the facility with the remaining participants. These cut‐off values allowed us to have adequate sample sizes in each group for regression modelling.

We used questionnaire data to identify members of groups known to have high HIV incidence (e.g. sex workers and MSM), and to classify participants into groups according to HIV risk factors related to PrEP eligibility as defined in the Kenya national guidelines [14]. Participants who reported their primary occupation as a sex worker and/or who reported engaging in transactional sex in the previous 12 weeks were classified as engaging in sex work/transactional sex. Having a partner living with HIV or an unknown HIV status was determined by having one or more partners living with HIV in the previous 12 weeks or if the participant indicated they “don't know” how many partners they had in the previous 12 weeks living with HIV. Participants who reported using condoms in <100% of sex acts (excluding oral sex) were considered as having inconsistent condom use. Alcohol and/or drug use in the previous 12 weeks was ascertained in individual questions and the results were combined into a single item related to alcohol and/or drug use. Participant responses to questions about STI diagnoses in the previous 12 weeks (i.e. gonorrhoea, syphilis, chlamydia, herpes, genital/anal warts or other STI) were combined into a single variable capturing recent STI diagnoses. Depression was assessed using the Patient Health Questionnaire (PHQ‐9) with scores categorized as none/minimal depression (0–4), mild depression (5–9) or moderate/severe depression (10–27) [20].

### 2.4 PEP/PrEP data collection and categorization

A CASI questionnaire about PEP/PrEP was administered at enrolment. Participants were first asked true/false questions to assess knowledge about HIV prevention options before being given definitions of PEP and PrEP. PEP was defined for participants as “A method for preventing HIV is called post exposure prophylaxis (PEP). PEP is a medication taken within 72 hours of exposure of HIV. The medication is taken by mouth every day for 28 days. When someone who does not have HIV is exposed to HIV through sex or injection drug use, PEP can work to keep the virus from establishing infection.” Immediately following the definition, on the same database screen, participants answered questions about PEP awareness, use, access and adherence. All participants were asked if they had ever heard of PEP, knew of a hospital or clinic where PEP could be accessed, or had ever used or attempted to access PEP. Participants who have ever used or attempted to access PEP were asked if they had ever had problems accessing PEP, how many courses of PEP they had taken and whether they completed their last course of PEP without any missed doses. Participants who indicated they had problems accessing PEP were asked about what problems were encountered. We categorized the number of PEP doses into 0, 1, ≥2 since Kenyan guidelines include recurrent PEP use as an eligibility criterion for PrEP [14]. Participants who indicated they had missed one or more doses during their last course of PEP were asked about the reasons for the missed dose(s) and how many doses were missed. All participants were given the opportunity to respond don't know or refuse to answer or skip any question. Don't know answers were recoded as no and refuse to answer responses were recoded as missing.

Similarly, PrEP was defined for participants before they answered questions related to PrEP awareness, use, access, adherence, and concerns and preferences regarding PrEP options. PrEP was defined for participants as “A method for preventing HIV is called pre‐exposure prophylaxis, or PrEP. PrEP is a medication taken to prevent HIV acquisition. Currently it is in the form of a pill taken every day or on demand when needed. When someone who does not have HIV is exposed to HIV through sex or injection drug use, PrEP can work to keep the virus from establishing infection. When taken consistently, daily oral PrEP has been shown to reduce the risk of HIV acquisition by up to 92% and up to 86% when taken on‐demand.” PrEP awareness and use was previously examined [19]; therefore, limited PrEP analyses are presented.

#### 2.4.1 Outcome definitions

We defined three PEP‐related outcomes to highlight implementation gaps. PEP awareness was defined by an affirmative response to the question, “Have you ever heard of PEP?” Among participants who heard of PEP, knowledge of where to access PEP was defined by an affirmative response to the question, “Do you know of a clinic or hospital in this area where PEP is available?” Lastly, among participants who heard of PEP, PEP use was defined by reporting one or more PEP courses in response to the question, “How many times have you taken a treatment course for PEP?” If a participant answered that they had taken PEP but previously indicated that they had never heard of PEP, the participant was reclassified as having heard of PEP. Participants who refused to answer or who were missing responses were classified as not reporting knowing where to access PEP or as not having reported PEP use.

### 2.5 Statistical analyses

The analytic population included all participants enrolled at the Kenyan sites who had a valid response to the question, “Have you ever heard of PEP?”. Descriptive analyses included counts and percentages for all variables and were stratified by outcome. We used Poisson regression with robust standard errors [21] to compute prevalence ratios (PRs) and adjusted prevalence ratios (aPRs) with 95% confidence intervals (CIs) to examine factors potentially associated with each PEP‐related outcome. For each outcome, we examined unadjusted models and an adjusted final model implementing purposeful variable selection [22, 23]. The purposeful variable selection process included three steps. First, we fit an adjusted model with all independent variables that were significant in their respective unadjusted models at the *p*<0.25 level. Second, using this initial adjusted model, we retained variables based on significance (*p*<0.1) in the adjusted model or confounding with other variables included in the adjusted model. A variable was retained based on confounding if the coefficient on any other independent variable changed by more than 20% when it was removed from the model. Variables not retained in this step were excluded from the final adjusted model. Finally, any independent variables not included in the initial adjusted model were added to the final model one at a time to determine if they became significant (*p*<0.15) in the presence of other independent variables. If any new variables were added to the model in this final stage, they were also assessed based on significance and confounding. Missingness in the independent variables was addressed using complete case analysis. *P*‐values <0.05 were considered statistically significant and *p*‐values <0.10 were considered suggestive of an association. All analyses were conducted using RStudio, version 2023.09.1 [24].

## 3 RESULTS

### 3.1 Participant characteristics

Of 485 people screened for study eligibility, 407 were enrolled, 399 attempted the PEP/PrEP questionnaire and 398 responded to the question “Have you ever heard of PEP?”. The analysed population was primarily female (*n* = 316, 79.4%; Table 1) with a median age of 22 years (IQR 19–24 years). In the 12 weeks prior to enrolment, the majority of participants reported inconsistent condom use (*n* = 282, 73.6%), partners with HIV or unknown HIV status (*n* = 278, 73.4%), transactional sex or sex work (*n* = 315, 81.0%), alcohol and/or drug use before sex (*n* = 233, 60.2%) and symptoms of depression (*n* = 228, 59.7%; Table 1). Forty‐three (10.9%) men reported sex with other men in the 12 weeks prior to enrolment; 21 of whom reported a primary occupation as a sex worker and/or engaging in transactional sex in the 12 weeks prior to enrolment.

**Table 1:** Participant characteristics, overall and by awareness, knowledge of where to access and ever having used HIV post‐exposure prophylaxis

		Heard of PEP		Knew where to access PEP^a^		Ever used PEP^a^	
	Overall	Yes	No	Yes	No	Yes	No
Characteristic	*n* (Column %) *N* = 398	*n* (Row %) *n* = 284 (71.4)	*n* (Row %) *n* = 114 (28.6)	*n* (Row %) *n* = 210 (73.9)	*n* (Row %) *n* = 74 (26.1)	*n* (Row %) *n* = 71 (25.0)	*n* (Row %) *n* = 213 (75.0)
Study site							
Kericho	181 (45.5)	120 (66.3)	61 (33.7)	73 (60.8)	47 (39.2)	24 (20.0)	96 (80.0)
Homa Bay	217 (54.5)	164 (75.6)	53 (24.4)	137 (83.5)	27 (16.5)	47 (28.7)	117 (71.3)
Sex							
Male	82 (20.6)	63 (76.8)	19 (23.2)	49 (77.8)	14 (22.2)	12 (19.0)	51 (81.0)
Female	316 (79.4)	221 (69.9)	95 (30.1)	161 (72.9)	60 (27.1)	59 (26.7)	162 (73.3)
Age							
≤24 years	317 (79.6)	224 (70.7)	93 (29.3)	157 (70.1)	67 (29.9)	46 (20.5)	178 (79.5)
>24 years	81 (20.4)	60 (74.1)	21 (25.9)	53 (88.3)	7 (11.7)	25 (41.7)	35 (58.3)
Marital status							
Not cohabitating or married	361 (90.9)	252 (69.8)	109 (30.2)	184 (73.0)	68 (27.0)	67 (26.6)	185 (73.4)
Cohabitating or married	36 (9.1)	31 (86.1)	5 (13.9)	25 (80.6)	6 (19.4)	4 (12.9)	27 (87.1)
Missing	1	1	0	1	0	0	1
Education level							
<12 years of education	209 (52.6)	139 (66.5)	70 (33.5)	87 (62.6)	52 (37.4)	34 (24.5)	105 (75.5)
≥12 years of education	188 (47.4)	144 (76.6)	44 (23.4)	122 (84.7)	22 (15.3)	37 (25.7)	107 (74.3)
Missing	1	1	0	1	0	0	1
Weekly household income^b^							
≤1000 Kenyan shillings	133 (33.5)	85 (63.9)	48 (36.1)	53 (62.4)	32 (37.6)	22 (25.9)	63 (74.1)
>1000 Kenyan shillings	264 (66.5)	198 (75.0)	66 (25.0)	156 (78.8)	42 (21.2)	49 (24.7)	149 (75.3)
Missing	1	1	0	1	0	0	1
Distance from facility							
≤3 km	95 (24.1)	76 (80.0)	19 (20.0)	67 (88.2)	9 (11.8)	22 (28.9)	54 (71.1)
>3 km	299 (75.9)	204 (68.2)	95 (31.8)	141 (69.1)	63 (30.9)	49 (24.0)	155 (76.0)
Missing	4	4	0	2	2	0	4
Ever heard of HIV pre‐exposure prophylaxis							
No	120 (30.4)	41 (34.2)	79 (65.8)	21 (51.2)	20 (48.8)	8 (19.5)	33 (80.5)
Yes	275 (69.6)	241 (87.6)	34 (12.4)	188 (78.0)	53 (22.0)	63 (26.1)	178 (73.9)
Missing	3	2	1	1	1	0	2
Inconsistent condom use^c^ ^,d^							
No	101 (26.4)	77 (76.2)	24 (23.8)	55 (71.4)	22 (28.6)	17 (22.1)	60 (77.9)
Yes	282 (73.6)	199 (70.6)	83 (29.4)	149 (74.9)	50 (25.1)	54 (27.1)	145 (72.9)
Missing	15	8	7	6	2	0	8
Partners with HIV or unknown HIV status^b^							
No	101 (26.6)	78 (77.2)	23 (22.8)	60 (76.9)	18 (23.1)	16 (20.5)	62 (79.5)
Yes	278 (73.4)	196 (70.5)	82 (29.5)	140 (71.4)	56 (28.6)	53 (27.0)	143 (73.0)
Missing	19	10	9	10	0	2	8
Transactional sex or sex work^b^							
No	74 (19.0)	59 (79.7)	15 (20.3)	52 (88.1)	7 (11.9)	10 (16.9)	49 (83.1)
Yes	315 (81.0)	220 (69.8)	95 (30.2)	155 (70.5)	65 (29.5)	61 (27.7)	159 (72.3)
Missing	9	5	4	3	2	0	5
Self‐reported sexually transmitted infection^b^							
No	339 (88.5)	243 (71.7)	96 (28.3)	174 (71.6)	69 (28.4)	61 (25.1)	182 (74.9)
Yes	44 (11.5)	32 (72.7)	12 (27.3)	28 (87.5)	4 (12.5)	10 (31.2)	22 (68.8)
Missing	15	9	6	8	1	0	9
Alcohol and/or drug use before sex^b^							
No	154 (39.8)	119 (77.3)	35 (22.7)	90 (75.6)	29 (24.4)	26 (21.8)	93 (78.2)
Yes	233 (60.2)	157 (67.4)	76 (32.6)	114 (72.6)	43 (27.4)	43 (27.4)	114 (72.6)
Missing	11	8	3	6	2	2	6
Depression^e^							
None/minimal	154 (40.3)	110 (71.4)	44 (28.6)	87 (79.1)	23 (20.9)	32 (29.1)	78 (70.9)
Mild	130 (34.0)	86 (66.2)	44 (33.8)	57 (66.3)	29 (33.7)	20 (23.3)	66 (76.7)
Moderate/severe	98 (25.7)	77 (78.6)	21 (21.4)	57 (74.0)	20 (26.0)	17 (22.1)	60 (77.9)
Missing	16	11	5	9	2	2	9

*Note*: At study entry, participants completed comprehensive socio‐behavioural questionnaires primarily by computer‐assisted self‐interview, including a dedicated questionnaire about HIV post‐exposure prophylaxis (PEP) and pre‐exposure prophylaxis (PrEP). Among 398 answered the question about whether they had ever heard of PEP. Among the 284 participants who had ever heard of PEP, 284 (100%) answered the question about knowing where to access PEP and all 284 (100%) were included in the PEP use outcome. For the overall study population, column percentages were calculated for each characteristic with missing values excluded from the denominator. Row percentages were calculated for each characteristic in analyses stratified by the outcomes of (1) having heard of PEP, (2) knowing where to access PEP and (3) ever having taken PEP.

Abbreviation: PEP, HIV post‐exposure prophylaxis.

^a^ Denominators for row percentages are the number of participants in the row who reported having heard of PrEP and had a non‐missing response to the outcome question (knows where to access PEP or has taken PEP).

^b^ 1000 Kenyan shillings was approximately equal to 7 USD at the time of survey administration.

^c^ Question asked about behaviours in the 12 weeks prior to enrolment.

^d^ Inconsistent condom use was defined as participants reporting condom use in fewer than 100% of all vaginal or anal sex.

^e^ None/minimal depression was defined as PHQ‐9 score 0–4, mild as 5–9 and moderate/severe as ≥10.

### 3.2 PEP/PrEP awareness, knowledge and access

**Figure 1:**
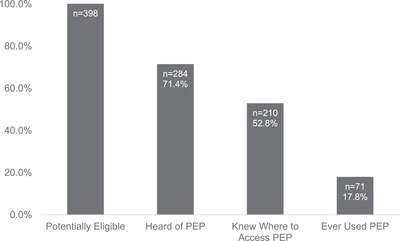
Number and percentage of participants who, at enrolment, are potentially eligible for PEP, heard of PEP, knew where to access PEP and ever used PEP. All percentages are out of the total number of participants who are potentially eligible for PEP (*n* = 398).

The majority of participants had heard of PEP (*n* = 284, 71.4%, Table 1 and Figure 1) and PrEP (*n* = 275, 69.6%). However, 114 (28.6%) participants had never heard of PEP, of whom 79 (69.3%) had also never heard of PrEP. One participant reported not having heard of PEP but also reported having taken one course of PEP; this participant was reclassified as having heard of PEP. While the majority of participants correctly indicated that there is a medicine that can be taken after sex for 28 days to prevent HIV (*n* = 295, 74.5%; Table 2) or that there is a pill that can be taken every day to prevent HIV (*n* = 270, 68.4%), a large proportion incorrectly thought a vaccine can prevent HIV (*n* = 193, 48.6%).

**Table 2:** HIV prevention knowledge, overall and by awareness, knowledge of where to access and ever having used HIV post‐exposure prophylaxis

		Heard of PEP		Knew where to access PEP		Ever used PEP	
	Total	Yes	No	Yes	No	Yes	No
Knowledge question	*n* (Column %) *N* = 398	*n* (Row %) *n* = 284	*n* (Row %) *n* = 114	*n* (Row %) *n* = 210	*n* (Row %) *n* = 74	*n* (Row %) *n* = 71	*n* (Row %) *n* = 213
True or False: There is a medicine that can be taken after sex for 28 days to prevent HIV infection.							
False	16 (4.0)	10 (62.5)	6 (37.5)	7 (70.0)	3 (30.0)	2 (20.0)	8 (80.0)
True	295 (74.5)	239 (81.0)	56 (19.0)	183 (76.6)	56 (23.4)	66 (27.6)	173 (72.4)
Don't know	85 (21.5)	33 (38.8)	52 (61.2)	19 (57.6)	14 (42.4)	3 (9.1)	30 (90.9)
Missing	2	2	0	1	1	0	2
True or False: There is a pill that can be taken every day to prevent HIV infection.							
False	38 (9.6)	30 (78.9)	8 (21.1)	23 (76.7)	7 (23.3)	7 (23.3)	23 (76.7)
True	270 (68.4)	213 (78.9)	57 (21.1)	164 (77.0)	49 (23.0)	61 (28.6)	152 (71.4)
Don't know	87 (22.0)	39 (44.8)	48 (55.2)	23 (59.0)	16 (41.0)	3 (7.7)	36 (92.3)
Missing	3	2	1	0	2	0	2
True or False: There are pills that can be taken to prevent HIV infection							
False	43 (10.9)	34 (79.1)	9 (20.9)	26 (76.5)	8 (23.5)	8 (23.5)	26 (76.5)
True	217 (54.9)	174 (80.2)	43 (19.8)	137 (78.7)	37 (21.3)	51 (29.3)	123 (70.7)
Don't know	135 (34.2)	75 (55.6)	60 (44.4)	46 (61.3)	29 (38.7)	12 (16.0)	63 (84.0)
Missing	3	1	2	1	0	0	1
True or False: There is a vaccine that can prevent HIV infection.							
False	68 (17.1)	53 (77.9)	15 (22.1)	41 (77.4)	12 (22.6)	9 (17.0)	44 (83.0)
True	193 (48.6)	153 (79.3)	40 (20.7)	121 (79.1)	32 (20.9)	49 (32.0)	104 (68.0)
Don't know	136 (34.3)	78 (57.4)	58 (42.6)	48 (61.5)	30 (38.5)	13 (16.7)	65 (83.3)
Missing	1	0	1	0	0	0	0

*Note*: At study entry, participants completed comprehensive socio‐behavioural questionnaires primarily by computer‐assisted self‐interview, including a series of true‐false questions to test participant knowledge about HIV post‐exposure prophylaxis (PEP) and pre‐exposure prophylaxis (PrEP). For the overall study population, column percentages were calculated for each response to a knowledge question with missing values excluded from the denominator. Row percentages were calculated for each response to a knowledge question in analyses stratified by the outcomes of (1) having heard of PEP (*n* = 398), (2) knowing where to access PEP among participants who have heard of PEP (*n* = 284) and (3) ever having taken PEP among participants who have heard of PEP (*n* = 284).

Abbreviation: PEP, HIV post‐exposure prophylaxis.

Two hundred ten (73.9%, Table 1) participants who had ever heard of PEP indicated they knew where PEP was available, and 71 (25.0%) participants who had heard of PEP reported having taken PEP. Among participants who had taken PEP, 33 (46.5%, Table 3) reported having taken two or more courses of PEP and 16 (22.5%) reported having problems accessing PEP. Participant‐reported access problems included PEP was not available (*n* = 5, 38.5%), PEP was too expensive (*n* = 4, 30.8%) and the participant was unable to consistently travel to pick up the medications (*n* = 4, 30.8%). One participant reported having taken or attempting to access PEP and also reported taking zero courses of PEP, yet this participant did not report problems accessing PEP. Sixteen (22.9%) participants who reported PEP use indicated they missed doses the last time they took a course of PEP. Reasons for missed dose(s) included no longer feeling the medication was needed (*n* = 5, 33.3%), receiving fewer than 28 doses (*n* = 4, 25.0%) and experiencing unwanted symptoms (*n* = 3, 20.0%).

**Table 3:** HIV post‐exposure prophylaxis use, access and adherence

	Participants who had ever used HIV PEP *N* = 71 (Column %)
Have you had problems accessing PEP?	
No	55 (77.5)
Yes	16 (22.5)
What problems did you have accessing PEP? (*n* = 16)	
Medication was not available	5 (38.5)
The medications are too expensive to consistently purchase	4 (30.8)
I am unable to consistently travel to pick up the medications	4 (30.8)
Missing	3
Number of courses of PEP	
1 course of PEP	38 (53.5)
2 or more courses of PEP	33 (46.5)
The last time you took PEP, did you complete the 28 doses of PEP without any missed doses?	
No	16 (22.9)
Yes	54 (77.1)
Missing	1
What was the reason for missed doses?	
Did not remember to take the medication	1 (6.7)
Stopped medication due to unwanted symptoms	3 (20.0)
No longer felt I needed the medication	5 (33.3)
Don't know	1 (6.7)
Participant defined other reasons for missed doses of PEP (*n* = 5)	5 (33.3)
Boyfriend threw the medications away after realizing it was PEP	1 (20.0)
Received fewer than 28 doses	4 (80.0)
Missing	1
Since completing PEP, how frequently do you find yourself using condoms?	
Never	2 (2.8)
More frequently	54 (76.1)
About the same	4 (5.6)
Less frequently	9 (12.7)
I'm still taking PEP	2 (2.8)

*Note*: Among 71 participants who reported that they had ever taken HIV post‐exposure prophylaxis (PEP), further questioning was conducted to characterize their PEP use, access to PEP and adherence to PEP.

Abbreviation: PEP, HIV post‐exposure prophylaxis.

### 3.3 Factors associated with PEP awareness and access

Factors associated with not having heard of PEP included completing fewer than 12 years of education (aPR 1.65 [95% CI 1.16–2.34], *p* = 0.005; Table 4) and marital status (Not cohabitating/married vs. cohabitating/married: aPR 2.81 [95% CI 1.11–7.08], *p* = 0.029). There was some evidence of an association between not having heard of PEP and inconsistent condom use in the previous 12 weeks (aPR 1.50 [95% CI 0.98–2.30], *p* = 0.063). In the initial purposeful variable selection process, both site and transactional sex were also retained in the final adjusted model. However, neither variable was significant even at the 0.25 level and each was only retained in the model due to a high degree of collinearity with each other, resulting in large changes to the coefficient on one variable when the other variable is excluded from the model, so both were excluded as non‐significant and the purposeful variable selection process was repeated only including the other 10 variables.

**Table 4:** Poisson regression analyses of factors potentially associated with never having heard of HIV post‐exposure prophylaxis

Characteristic	Never heard of PEP^a^ *n* (row %)	Unadjusted prevalence ratio (95% CI)	*p*‐value	Adjusted prevalence ratio (95% CI)	*p*‐value
Study site					
Kericho	57 (34.5)	**1** **.48 (1.06**–**2.07)**	**0.022**		
Homa Bay	43 (23.4)	Reference			
Sex					
Male	13 (19.4)	Reference			
Female	87 (30.9)	1.59 (0.95–2.67)	0.080		
Age					
≤24 years	81 (29.3)	1.13 (0.74–1.73)	0.582		
>24 years	19 (26.0)	Reference			
Marital status					
Not cohabitating or married	96 (30.5)	**2.59 (1.02**–**6.60)**	**0.046**	**2.81 (1.11**–**7.08)**	**0.029**
Cohabitating or married	4 (11.8)	Reference		Reference	
Education level					
**<**12 years of education	66 (34.6)	**1.61 (1.12**–**2.29)**	**0.009**	**1.65 (1.16**–**2.34)**	**0.005**
≥12 years of education	34 (21.5)	Reference		Reference	
Weekly household income					
≤1000 Kenyan shillings	38 (33.6)	1.28 (0.91–1.79)	0.15		
>1000 Kenyan shillings	62 (26.3)	Reference			
Distance from healthcare facility					
≤3 km	14 (17.9)	Reference			
>3 km	86 (31.7)	**1.77 (1.07**–**2.93)**	**0.027**		
Inconsistent condom use^b^					
No	19 (22.6)	Reference		Reference	
Yes	81 (30.6)	1.35 (0.87–2.09)	0.175	1.50 (0.98–2.30)	0.063
Partners with HIV or unknown HIV status^b^					
No	22 (23.7)	Reference			
Yes	78 (30.5)	1.29 (0.86–1.94)	0.226		
Transactional sex or sex work^b^					
No	11 (16.7)	Reference			
Yes	89 (31.4)	**1.89 (1.07**–**3.32)**	**0.028**		
Self‐reported sexually transmitted infection^b^					
No	89 (29.0)	Reference			
Yes	11 (26.2)	0.90 (0.53–1.55)	0.711		
Alcohol and/or drug use before sex^b^					
No	29 (21.5)	Reference			
Yes	71 (33.2)	**1.54 (1.06**–**2.25)**	**0.023**		
Depression^c^					
None/minimal	40 (27.6)	Reference			
Mild	42 (35.6)	1.29 (0.90–1.85)	0.163		
Moderate or severe	18 (20.9)	0.76 (0.47–1.24)	0.268		

*Note*: Poisson regression with robust standard errors was used to calculate prevalence ratios and 95% confidence intervals for the association between pre‐specified participant characteristics and never having heard of PEP. Unadjusted modelling was performed for each characteristic of interest and purposeful variable selection was used to identify participant characteristics for inclusion in the final adjusted model. Results significant at the 5% level are presented in bold font.

Abbreviations: CI, confidence interval; PEP, HIV post‐exposure prophylaxis.

^a^ Since complete case analysis (*n* = 349) was used to deal with missingness, the counts and percentages will not match those in Table 1.

^b^ Question asked about behaviours in the 12 weeks prior to enrolment.

^c^ None/minimal depression was defined as PHQ‐9 score 0–4, mild as 5–9 and moderate/severe as ≥10.

Among participants who reported hearing of PEP, factors associated with not knowing where to access PEP included completing fewer than 12 years of education (aPR 2.20 [95% CI 1.37–3.54], *p* = 0.001; Table 5) and depression (mild aPR 1.86 [95% CI 1.17–2.96], *p* = 0.009; moderate or severe aPR 1.84 [95% CI 1.09–3.09], *p* = 0.021). There was some evidence that participants with a self‐reported STI in the previous 12 weeks were more likely to know where to access PEP (aPR 0.41 [95% CI 0.15–1.13], *p* = 0.086).

**Table 5:** Poisson regression analyses of factors potentially associated with not knowing where to access HIV post‐exposure prophylaxis, among participants who had ever heard of it

Characteristic	Does not know where to access PEP^a^ *n* (row %)	Unadjusted prevalence ratio (95% CI)	*p*‐value	Adjusted prevalence ratio (95% CI)	*p*‐value
Study site					
Kericho	42 (38.9)	**2.38 (1.53**–**3.71)**	**<0.001**	1.40 (0.84–2.35)	0.198
Homa Bay	23 (16.3)	Reference		Reference	
Sex					
Male	11 (20.4)	Reference			
Female	54 (27.7)	1.36 (0.77–2.41)	0.294		
Age					
≤24 years	58 (29.7)	**2.29 (1.11**–**4.73)**	**0.025**	1.51 (0.72–3.18)	0.275
>24 years	7 (13.0)	Reference		Reference	
Marital status					
Not cohabitating or married	59 (26.9)	1.35 (0.64–2.85)	0.435		
Cohabitating or married	6 (20.0)	Reference			
Education level					
**<**12 years of education	47 (37.6)	**2.59 (1.60**–**4.20)**	**<0.001**	**2.20 (1.37**–**3.54)**	**0.001**
≥12 years of education	18 (14.5)	Reference		Reference	
Weekly household income					
≤1000 Kenyan shillings	28 (37.3)	**1.76 (1.17**–**2.64)**	**0.007**		
>1000 Kenyan shillings	37 (21.3)	Reference			
Distance from healthcare facility					
≤3 km	7 (10.9)	Reference		Reference	
>3 km	58 (31.4)	**2.87 (1.38**–**5.95)**	**0.005**	1.85 (0.88–3.93)	0.107
Inconsistent condom use^b^					
No	18 (27.7)	Reference			
Yes	47 (25.5)	0.92 (0.58–1.47)	0.733		
Partners with HIV or unknown HIV status^b^					
No	16 (22.5)	Reference			
Yes	49 (27.5)	1.22 (0.75–2.00)	0.426		
Transactional sex or sex work^b^					
No	7 (12.7)	Reference			
Yes	58 (29.9)	**2.35 (1.14**–**4.85)**	**0.021**		
Self‐reported sexually transmitted infection^b^					
No	62 (28.4)	Reference		Reference	
Yes	3 (9.7)	0.34 (0.11–1.02)	0.054	0.41 (0.15–1.13)	0.086
Alcohol and/or drug use before sex^b^					
No	25 (23.6)	Reference			
Yes	40 (28.0)	1.19 (0.77–1.83)	0.439		
Depression^c^					
None/minimal	20 (19.0)	Reference		Reference	
Mild	27 (35.5)	**1.87 (1.13**–**3.07)**	**0.014**	**1.86 (1.17**–**2.96)**	**0.009**
Moderate or severe	18 (26.5)	1.39 (0.79–2.43)	0.249	**1.84 (1.09**–**3.09)**	**0.021**

*Note*: Poisson regression with robust standard errors was used to calculate prevalence ratios and 95% confidence intervals for the association between pre‐specified participant characteristics and not knowing where to access PEP, among those who have heard of PEP. Unadjusted modelling was performed for each characteristic of interest and purposeful variable selection was used to identify participant characteristics for inclusion in the final adjusted model. Results significant at the 5% level are presented in bold font.

Abbreviations: CI, confidence interval; PEP, HIV post‐exposure prophylaxis.

^a^ Since complete case analysis (*n* = 249) was used to deal with missingness, the counts and percentages will not match those in Table 1.

^b^ Question asked about behaviours in the 12 weeks prior to enrolment.

^c^ None/minimal depression was defined as PHQ‐9 score 0–4, mild as 5–9 and moderate/severe as ≥10.

Factors associated with being less likely to have used PEP among participants who reported hearing of PEP included age 24 years or younger (aPR 1.34 [95% CI 1.02–1.75], *p* = 0.032), marital status (Not cohabitating/married vs. cohabitating/married aPR 0.75 [95% CI 0.60–0.95], *p* = 0.016; Table 6) and engagement in transactional sex or sex work (aPR 0.77 [95% CI 0.63–0.94], *p* = 0.009). Though they were not significantly associated with having used PEP in the adjusted model, study site, distance from healthcare facility, sexual partners with HIV or unknown HIV status in the previous 12 weeks and depression were also included in the final model due to confounding with other independent variables included in the model.

**Table 6:** Poisson regression analyses of factors potentially associated with never having taken HIV post‐exposure prophylaxis, among participants who had ever heard of it

Characteristic	Never used PEP^a^ *n* (row %)	Unadjusted prevalence ratio (95% CI)	*p*‐value	Adjusted prevalence ratio (95% CI)	*p*‐value
Study site					
Kericho	85 (78.7)	1.13 (0.98–1.31)	0.097	1.21 (0.96–1.53)	0.098
Homa Bay	98 (69.5)	Reference		Reference	
Sex					
Male	44 (81.5)	Reference			
Female	139 (71.3)	0.87 (0.75–1.02)	0.091		
Age					
≤24 years	151 (77.4)	**1.31 (1.03**–**1.65)**	**0.025**	**1.35 (1.04**–**1.76)**	**0.023**
>24 years	32 (59.3)	Reference		Reference	
Marital status					
Not cohabitating or married	157 (71.7)	**0.83 (0.70**–**0.97)**	**0.023**	**0.75 (0.60**–**0.95)**	**0.015**
Cohabitating or married	26 (86.7)	Reference		Reference	
Education level					
**<**12 years of education	93 (74.4)	1.03 (0.88–1.19)	0.745		
≥12 years of education	90 (72.6)	Reference			
Weekly household income					
≤1000 Kenyan shillings	53 (70.7)	0.95 (0.80–1.12)	0.520		
>1000 Kenyan shillings	130 (74.7)	Reference			
Distance from healthcare facility					
≤3 km	42 (65.6)	Reference		Reference	
>3 km	141 (76.2)	1.16 (0.96–1.41)	0.132	1.14 (0.92–1.40)	0.235
Inconsistent condom use^b^					
No	50 (76.9)	Reference			
Yes	133 (72.3)	0.94 (0.80–1.10)	0.447		
Partners with HIV or unknown HIV status^b^					
No	56 (78.9)	Reference		Reference	
Yes	127 (71.3)	0.90 (0.78–1.05)	0.197	0.88 (0.71–1.09)	0.239
Transactional sex or sex work^b^					
No	46 (83.6)	Reference		Reference	
Yes	137 (70.6)	**0.84 (0.73**–**0.98)**	**0.025**	**0.78 (0.64**–**0.94)**	**0.010**
Self‐reported sexually transmitted infection^b^					
No	162 (74.3)	Reference			
Yes	21 (67.7)	0.91 (0.71–1.18)	0.477		
Alcohol and/or drug use before sex^b^					
No	81 (76.4)	Reference			
Yes	102 (71.3)	0.93 (0.80–1.08)	0.363		
Depression^c^					
None/minimal	73 (69.5)	Reference		Reference	
Mild	59 (77.6)	1.12 (0.94–1.33)	0.217	1.16 (0.98–1.37)	0.085
Moderate or severe	51 (75.0)	1.08 (0.90–1.30)	0.426	1.14 (0.95–1.36)	0.168

*Note*: Poisson regression with robust standard errors was used to calculate prevalence ratios and 95% confidence intervals for the association between pre‐specified participant characteristics and never having taken PEP, among those who have heard of PEP. Unadjusted modelling was performed for each characteristic of interest and purposeful variable selection was used to identify participant characteristics for inclusion in the final adjusted model. Results significant at the 5% level are presented in bold font.

Abbreviations: CI, confidence interval; PEP, HIV post‐exposure prophylaxis.

^a^ Since complete case analysis (*n* = 249) was used to deal with missingness, the counts and percentages will not match those in Table 1.

^b^ Question asked about behaviours in the 12 weeks prior to enrolment.

^c^ None/minimal depression was defined as PHQ‐9 score 0–4, mild as 5–9 and moderate/severe as ≥10.

Sensitivity analyses were conducted to determine the impact of outcome definitions and missingness on the results. There were no significant changes in the results based on either the inclusion/exclusion of missing responses in the analyses or if PEP use was defined based upon “Have you ever taken or attempted to access PEP?” or “How many times have you taken a treatment course of PEP?”.

## 4 DISCUSSION

Despite enrolling participants who reported behaviours associated with vulnerability to HIV, we found that more than a quarter of participants had never heard of PEP and many more did not know where to access it. While PEP awareness in this study was suboptimal, it was higher than expected considering that previous studies had reported PEP awareness in Africa between 20% and 56.7% [25, 26, 27, 28]. Our study was conducted in an area that has hosted many prior HIV‐related studies, particularly at the Kericho site, so the enrolled population may have been exposed to increased HIV prevention messaging as a result. Regardless, substantial gaps in PEP awareness were identified. Furthermore, there were substantial gaps in knowledge about PEP/PrEP options with almost half of participants thinking there is an existing HIV vaccine and more than 25% unaware that PEP was available. When considering participants who were aware of PEP, more than 40% did not know where it was available and more than 20% of participants who tried to access PEP reported having experienced access problems.

Previous work has found that barriers to PEP implementation include low awareness of PEP as an HIV prevention tool among people vulnerable to HIV [29, 30], difficulties in the healthcare system to handle non‐occupational PEP requests, lack of confidence among healthcare providers to prescribe PEP [29], concerns about confidentiality and/or discomfort disclosing HIV status [29, 30], potential side effects and lack of access to PEP [29]. In spite of Kenyan guidelines for PrEP indicating that recurrent use of PEP is one eligibility criterion for PrEP [14], we found that participants lack knowledge of PEP as a prevention tool, consistent with previous findings. Other findings consistent with previous work include low PEP awareness and a lack of knowledge about where to access PEP. Furthermore, our finding that some participants were not given a full course of PEP is an indication supporting previous work that providers may not be confident prescribing PEP. Provider confidence in prescribing PEP is not well‐documented, especially in sub‐Saharan Africa. However, several studies surveying U.S. providers report low provider confidence in prescribing PEP and/or PrEP, regardless of their years of experience or age [31, 32, 33]. Having lower education levels was associated with both a lack of PEP awareness and a lack of knowledge of where to access PEP among participants who had heard of PEP. Our finding that having lower education levels was associated with a lack of PEP awareness is consistent with this previous research from geographically diverse study locations [34, 35, 36, 37, 38]. Few studies have examined the association between socio‐demographic characteristics or behaviours and knowledge of where to access PEP. However, previous work has found that knowledge of where to access PEP is low [39, 40]. Among participants who were aware of PEP, being younger and being married were associated with an increased prevalence of never taking PEP. These gaps represent opportunities for improved programming related to PEP as a biomedical HIV prevention tool for participants with occasional exposure to HIV, highlighting the importance of targeted messaging for younger people and people with lower socio‐economic status.

The relationship between depression and HIV prevention/care engagement is not well understood. While some studies have reported that depression is not associated with HIV care and prevention‐related outcomes [41, 42], many studies report that experiencing depressive symptoms is associated with lower adherence to HIV medication regimens, lower engagement in HIV‐related healthcare, and worse health outcomes in people living with HIV and with reduced adherence to HIV prevention regimens and an increased likelihood of acquisition in people living without HIV [43, 44, 45, 46, 47, 48, 49, 50, 51]. In our study, depression was associated with both never taking PEP and not knowing where to access PEP among participants who had heard of PEP. While depressive symptoms may be associated with any number of factors related or unrelated to a possible HIV exposure, this finding highlights the importance of addressing mental health in the context of HIV prevention for people who engage in behaviours that may increase the risk of HIV exposure.

Enrolment of participants with behavioural vulnerability to HIV allowed us to measure PEP‐related outcomes in a population that was likely eligible for PEP/PrEP. However, these analyses should be interpreted in the context of several limitations. The MOCHI study was powered to study HIV incidence and may have been underpowered to assess all potential factors associated with PEP‐related outcomes. Future work in larger cohorts should examine these relationships in detail. Questionnaires assessed participant demographic characteristics at enrolment and behavioural characteristics within the previous 12 weeks, but questions about PEP use did not identify a time frame, complicating the analysis and interpretation of PEP use and eligibility. Participants were provided a definition of PEP immediately before being asked whether they had ever heard of PEP; while the definition was intended to clarify the question, some participants may have reported hearing of PEP based only on the definition they had just been provided. Additionally, the definitions of PEP and PrEP may have included terms that were unfamiliar or confusing to participants. Lastly, questionnaire data may be susceptible to biases related to recall, self‐report and social desirability.

## 5 CONCLUSIONS

Despite enrolling people behaviourally vulnerable to HIV, we identified substantial gaps in PEP awareness, knowledge and uptake. These gaps included a lack of awareness of PEP and PrEP, a lack of knowledge about where to access PEP and problems accessing PEP. Participants with lower education levels have lower awareness of PEP, whereas younger participants have lower PEP utilization than older participants, and participants with depression have both lower PEP utilization and knowledge of where to access PEP than participants without depression. Therefore, targeting outreach efforts to populations with lower education levels and including mental healthcare in HIV prevention programming may help alleviate some of the PEP awareness and uptake barriers.

## COMPETING INTERESTS

The authors have no conflicts of interest to disclose.

## AUTHORS’ CONTRIBUTIONS

MLR, GS, JK and TAC developed the concept and designed the analysis plan with input at various points from AY, JG, HQ, BG, MCT, RB, DL, CA, CC, MY and FS. CA, RB and DL collected and managed the data at the site. GS, AY and NB conducted the statistical analyses. GS wrote the first draft of the manuscript with critical input from all co‐authors. All authors edited the manuscript and provided final approval for submission.

## FUNDING

This work was supported by agreements (W81XWH‐18‐2‐0040; HT9425‐24‐3‐0004) between the Henry M. Jackson Foundation for the Advancement of Military Medicine, Inc. and the U.S. Department of Defense. This research was funded, in part, by the U.S. National Institute of Allergy and Infectious Diseases (AAI20052001). The investigators have adhered to the policies for the protection of human research participants as prescribed in AR 70–25.

## DISCLAIMER

Material has been reviewed by the Walter Reed Army Institute of Research. There is no objection to its presentation and/or publication. The opinions or assertions contained herein are the private views of the author, and are not to be construed as official, or as reflecting true views of the Department of the Army or the Department of Defense.

## Data Availability

The data that support the findings of this study are available on request from the corresponding author. The data are not publicly available due to privacy or ethical restrictions.
